# The Prebiotic Effects of Oats on Blood Lipids, Gut Microbiota, and Short-Chain Fatty Acids in Mildly Hypercholesterolemic Subjects Compared With Rice: A Randomized, Controlled Trial

**DOI:** 10.3389/fimmu.2021.787797

**Published:** 2021-12-09

**Authors:** Dengfeng Xu, Meiyuan Feng, YiFang Chu, Shaokang Wang, Varsha Shete, Kieran M. Tuohy, Feng Liu, Xirui Zhou, Alison Kamil, Da Pan, Hechun Liu, Xian Yang, Chao Yang, Baoli Zhu, Na Lv, Qian Xiong, Xin Wang, Jianqin Sun, Guiju Sun, Yuexin Yang

**Affiliations:** ^1^ Key Laboratory of Environmental Medicine and Engineering of Ministry of Education, Department of Nutrition and Food Hygiene, School of Public Health, Southeast University, Nanjing, China; ^2^ Department of R&D Life Science, PepsiCo, Inc., Shanghai, China; ^3^ Department of R&D Life Science, PepsiCo, Inc., Barrington, IL, United States; ^4^ Department of Food Quality and Nutrition, Research Innovation Centre, Fondazione Edmund Mach, Trento, Italy; ^5^ CAS Key Laboratory of Pathogenic Microbiology and Immunology, Institute of Microbiology, Chinese Academy of Sciences, Beijing, China; ^6^ Department of Nutrition and Functional Food Research, Beijing Research Institute for Nutritional Resources, Beijing, China; ^7^ Department of Clinical Nutrition, Huadong Hospital Affiliated to Fudan University, Shanghai, China; ^8^ National Institute for Nutrition and Health, Center for Disease Control and Prevention, Beijing, China

**Keywords:** oat, β-glucan, polyphenol, cholesterol, microbiota, short-chain fatty acids

## Abstract

Phytochemicals derived from oats are reported to possess a beneficial effect on modulating dyslipidemia, specifically on lowering total and LDL cholesterol. However, deeper insights into its mechanism remain unclear. In this randomized controlled study, we assigned 210 mildly hypercholesterolemic subjects from three study centers across China (Beijing, Nanjing, and Shanghai) to consume 80 g of oats or rice daily for 45 days. Plasma lipid profiles, short chain fatty acids (SCFAs), and fecal microbiota were measured. The results showed that total cholesterol (TC) and non-high-density lipoprotein cholesterol (non-HDL-C) decreased significantly with both oats and rice intake after 30 and 45 days. The reduction in TC and non-HDL-C was greater in the participants consuming oats compared with rice at day 45 (*p* = 0.011 and 0.049, respectively). Oat consumption significantly increased the abundance of *Akkermansia muciniphila* and *Roseburia*, and the relative abundance of *Dialister*, *Butyrivibrio*, and *Paraprevotella*, and decreased unclassified *f-Sutterellaceae.* In the oat group, *Bifidobacterium* abundance was negatively correlated with LDL-C (*p* = 0.01, *r* = −0.31) and, TC and LDL-C were negatively correlated to *Faecalibacterium prausnitzii* (*p* = 0.02, *r* = −0.29; *p* = 0.03, *r* = −0.27, respectively). *Enterobacteriaceae*, *Roseburia*, and *Faecalibacterium prausnitzii* were positively correlated with plasma butyric acid and valeric acid concentrations and negatively correlated to isobutyric acid. HDL-C was negatively correlated with valeric acid (*p* = 0.02, *r* = −0.25) and total triglyceride (TG) was positively correlated to isovaleric acid (*p* = 0.03, *r* = 0.23). Taken together, oats consumption significantly reduced TC and LDL-C, and also mediated a prebiotic effect on gut microbiome. *Akkermansia muciniphila*, *Roseburia*, *Bifidobacterium*, and *Faecalibacterium prausnitzii*, and plasma SCFA correlated with oat-induced changes in plasma lipids, suggesting prebiotic activity of oats to modulate gut microbiome could contribute towards its cholesterol-lowering effect.

## 1 Introduction

Coronary heart disease (CHD) is a major cause of death across the world (1), as well as in China (2), and hypercholesterolemia is recognized as an important risk factor for CHD (3). Oats and oat products have demonstrated an ability to reduce cholesterol, with recent meta-analysis confirming that oat β-glucan having a significant lowering effect on low-density lipoprotein cholesterol (LDL-C), non-high-density lipoprotein cholesterol (non-HDL-C), and other markers of CHD (4). Similarly, a meta-analysis by Tiwari and Cummins shown an inverse relation between the consumption of β-glucan and the levels of total cholesterol (TC) and LDL-C; in addition, the results of this meta-analysis also indicated a dose-response relationship between β-glucan and cholesterol-lowering effect (5).

Oat β-glucan is a part of the larger family of mixed-linkage β-glucans, with a structure of linear polymers of β-anhydroglucopyranosyl units connected by mainly 1→3 and 1→4 linkages (6). It is a soluble fiber with gel-forming properties, which increases its viscosity upon ingestion in the small intestine, and this property aids β-glucan to bind bile acids and possibly cholesterol in the small intestine, and hence reduce the absorption of bile acids (BAs) and cholesterol from the gut (7, 8). This then increases fecal excretion of BAs and cholesterol (6, 9). Since the total BAs pool is tightly regulated, loss of BAs in feces drives hepatic BA synthesis and sequestration of circulating cholesterol. This phenomenon has been proposed as the main mechanism underpinning the cholesterol-lowering effect of oat β-glucan (10).

High heterogeneity in LDL-C lowering effect of oats has been reported across dietary interventions (11, 12). Such heterogeneity may be due to differences in test products but also high interindividual variation in response among subjects. The cholesterol-lowering effect of oats has been observed to be modified by host genotype, specifically cytochrome P450 family 7 subfamily A member 1 gene rs3808607 genotype in hypercholesterolemic individuals. It has been seen that individuals with TT genotype exhibited higher reponsiveness in reducing LDL-C than G allele carriers (13). Similarly, human gut microbiota is also modulated by dietary factors such as fiber and polyphenols, and in turn, plays an important role in degradation of complex plant molecules which escape digestion in the stomach and small intestine (14, 15). Gut microbiome has been shown to differ according to geography, and this represents an important confounding factor driven by population-specific diets and lifestyle (16, 17). Indeed, Andersson et al. suggested that gut microbiota composition and BA metabolism may influence the cholesterol-lowering response to oats in two strains of the same laboratory mouse line divergent for oat-induced cholesterol lowering on a high-fat diet (18). Additionally, by interacting with the immune system and through production of bioactive compounds like short chain fatty acids (SCFAs, such as acetate, propionate, and butyrate), gut microbiota appears to play an important role in individual response to foods or diets which influence host metabolism and disease risk (19). Recent studies conducted by Connolly et al. have explored the influence of whole grain oat granola on lowering cholesterol levels in hypercholesterolemic subjects and found a significant decrease in TC levels and LDL-C after consuming 45 g whole grain oat granola for 6 weeks; they also observed a significant increase in the abundance of *Bifidobacteria* and *Lactobacilli* within the fecal microbiota of subjects following oat consumption compared within the control nonwhole grain breakfast cereal (20). The possibility therefore exists, that oat and barley induced changes within the gut microbiota, for example, through SCFA production, could contribute to the cholesterol-lowering effects of oats. However, few studies have coanalyzed oat-induced changes in cholesterol and changes in gut microbiota. Moreover, fecal samples were usually used in most of the previous studies to detect the level of SCFAs (20–22), which may not accurately reflect the circulating level of SCFAs in the body.

The aim of this study was to explore the relationship between blood lipids, gut microbiota, and plasma SCFAs in a Chinese population with mild hypercholesterolemia by applying metagenomic and metabolomic approaches. Our hypothesis was that consuming 80 g of oats per day for 45 days would improve blood lipid and modulate the gut microbiota, with a concomitant increase in plasma SCFA concentrations, providing a plausible link between oat-induced microbiota modulation and lowering of LDL-C in hypercholesterolemic Chinese subjects. This study would therefore also confirm the prebiotic nature of oats and provide new insight into the putative gut microbiota related contribution towards the cholesterol-lowering effect of oats.

## 2 Methods

### 2.1 Participants (Including Sample Size Calculation)

The study was registered in China Clinical Trials (www.chictr.org.cn) and was given a favorable ethics evaluation and approved by the China Ethics Committee of Registering Clinical Trials (ChiECRCT-20180139) and was also compliant with Declaration of Helsinki guidelines. Written informed consent was obtained from all volunteers.

The subjects with mild hypercholesterolemia in local hospital and communities were recruited (*n* = 210). The criteria of diagnosing mild hypercholesterolemia were described previously (23). Briefly, the eligibility criteria were as follows (1): participants 18 to 65 years old with body mass index (BMI) <28 kg/m^2^ (2); plasma TC values ≥5.18 mmol/L but ≤6.21 mmol/L, and total triglyceride (TG) ≤2.25 mmol/L (3); no diagnoses of serious kidney, liver, or digestive tract disease, or diabetes or other metabolic disease (4); no use within the previous 3 months of relevant medicines characterized as having cholesterol-lowing effects. The exclusion criteria were as follows (1): pregnancy or lactation (2); daily intake of oats or other foods rich in β-glucan for the last 6 months (3); history of heavy smoking or alcoholism (4); current use of weight loss diets; and (5) poor compliance.

The primary objective was a change in plasma TC levels. Secondary objectives included circulating lipid profiles, SCFAs, and fecal microbiota composition. The sample size for this study was estimated for a two-group parallel superiority randomized control trial using the below equation (24):


$$n_{1}=n_{2}=2{\left[\frac{\left(\mu_{a}+\mu_{\beta }\right)}{\delta /\sigma }\right]}^{2}+\frac{1}{4}\mu_{\alpha }^{2},$$

In which, *μ*_α_ and *μ*_β_ were designated as 1.96 and 0.842, respectively; *δ* and *σ* were the net mean changes in primary outcomes and the standard deviation (SD) values for the two groups, respectively. The change in TC levels was the primary outcome variable, assuming that the net mean change was 0.23 and the SD was 0.56 (7). Therefore, the per group sample size was calculated to be about 93. To account for a 10% dropout rate, 105 volunteers per group were targeted for recruitment.

### 2.2 Study Design (Including Randomization and Blinding)

A multicenter randomized, controlled, and parallel-designed trial in Beijing, Nanjing, and Shanghai, China was conducted. Prior to the trial, a screening visit (including the anthropometric measurements using a stadiometer and scale, plasma cholesterol measurements) was conducted to evaluate the eligibility for enrolment of each participant.

The eligible participants were randomly assigned to the experimental group (oats) or the control group (rice), which were balanced by sex, age (<50 or ≥50 years), and BMI (<24 or ≥24 kg/m^2^) based on a stratified block design using Microsoft Excel. The study was conducted in a single-blind manner. Subjects in each group were required to consume a total of 80 g oat containing 3.0 g β-glucan and 56.8 mg polyphenol or rice daily for 45 days at the same time maintaining their habitual diet. All test samples were provided by a Quaker Oats manufacturing facility (PepsiCo, Inc., Shanghai, China). The nutrients of the test foods are shown in **Supplementary Table S1**. The test foods were regularly provided to participants once every week. To ensure high compliance in the test population, participants were excluded if they did not consume the test foods for 2 or more days per week or for 2 consecutive days. Participants could communicate potential adverse effects and relevant concerns with the investigator on a weekly basis. The blood samples were collected from participants at Days 0, 30, and 45 (end of the study). The fecal samples were collected from participants at Days 0 and 45. The anthropometric measurements were conducted at Day 0.

### 2.3 Outcome Measures

#### 2.3.1 Blood Sample Cllection and Analysis (for Cholesterol and Other Parameter Analysis)

Blood was collected *via* venipuncture into sodium citrate containing tubes after an overnight fast on Days 0, 30, and 45 on intervention (sodium citrate: blood ratio was 1:9, provided by Shijiazhuang Kang Weishi Medical Instrument Co., Ltd. China). Blood samples were centrifuged at 1,500×*g* for 15 min at 4°C (L-550, Hunan Xiangyi Centrifuge Instrument Co., Ltd. China) to collect plasma and stored in 2 ml cryogenic tube (Corning 430659, USA) at −80°C until analysis.

#### 2.3.2 Blood Lipid Analysis

TC, TG, LDL-C, and HDL-C were measured in plasma using an automatic biochemical analyzer (Beckman, DxC800, USA) and commercial kits (BioSino Bio-Technology & Science Inc, Beijing, China) according to manufacturer’s instructions. The non-HDL-C was calculated by using TC minus HDL-C.

#### 2.3.3 Plasma SCFAs Analysis

SCFAs were analyzed by using plasma samples based on the methods of Zhao et al. with some modification (25). The 0.15-ml sample was added into 1.5 ml Eppendorf tube with 0.05 ml 50%H_2_SO_4_ and 0.2 ml of 2-methylvaleric acid (25 mg/L stock in methyltert-butylether) as internal standard. The sample was then vortexed for 30 s, followed by 10 min oscillations and 10 min ultrasound treatment. After this, the supernatant was collected after 10 min of 12,000 rpm centrifugation and kept at −20°C for 30 min. The supernatant was transferred into a clean 2 ml glass vial for gas-chromatography mass spectrometry (GC-MS) analysis.

GC-MS was used for SCFA analysis, targeting 7 SCFAs which were acetic acid, acetic acid, isobutyric acid, butyric acid, isovaleric acid, valeric acid, and hexanoic acid. GC-MS analysis was performed using an Agilent 7890B gas chromatograph system coupled with Agilent 5977B mass spectrometer. The system utilized a DB-FFAP capillary column (15 m × 250 μm × 0.25 μm). A 1-μl aliquot of the analyte was injected in split mode (5:1). Helium was used as the carrier gas, the front inlet purge flow was 3 ml min^−1^, and the gas flow rate through the column was 1 ml min^−1^. The initial temperature was kept at 80°C for 1 min, then raised to 180°C at a rate of 10°C min^−1^, kept 1 min, then kept for 5 min at 240°C at a rate of 20°C min^−1^. The injection, transfer line, quad, and ion source temperatures were 240°C, 240°C, 230°C, and 150°C. The energy was −70eV in electron impact mode. The mass spectrometry data were acquired in scan mode with the *m*/*z* range of 33–150 after a solvent delay of 2.5 min.

#### 2.3.4 Microbiota Analysis

##### 2.3.4.1 Fecal Sample Collection for DNA Extraction

In order to obtain representative fecal samples, at days 0 and 45, each site had a professional and well-trained researcher whose responsibility was collecting the fecal samples into the specific tubes containing DNA preservatives according to standard procedures for the purpose of avoiding the degradation of bacterial DNA (the tubes were provided by Guangdong Longsee Biomedical Co., Ltd. Guangdong, China), then the sample tubes were snap frozen in liquid nitrogen within minutes of donation and stored at −80°C until DNA extraction. Prior to the microbiota detection, according to the manufacturer, approximately 250 mg feces was taken from every sample (a total of 177 samples in both Shanghai and Nanjing sites) for DNA extraction by using the recommended kit (QIAamp, Powerfecal Pro DNA Kit, Qiagen, Germany).

##### 2.3.4.2 Real-Time Quantitative Polymerase Chain Reaction (RT-qPCR)

Real time quantitative PCR was applied to examine the changes of 8 bacteria of interest based on previous studies with oats and prebiotic fibers. The 8 targeted bacteria were *Bifidobacterium* (genus), *Lactobacillus* (genus), *Akkermansiaceae* (species), *Roseburia* (genus), *Enterobacteriaceae* (family), *Bacteroidaceae* (genus), *Faecalibacterium prausnitzii* (species), and *Clostridium perfringens* (species).

The abundance of targeted bacteria was measured by 16S rDNA gene using TaqMan Real-Time qPCR in an ABI 7500 Real time KaPa enzyme PCR system (Institute of Microbiology, Chinese Academy of Sciences, Beijing, China). The specific primers and enzyme system are shown in **Supplementary Tables 2, 3**). Briefly, the samples were taken from freezer and stored on the ice, mixed with reagents evenly, and then transferred to qPCR plate and shaken evenly. The prepared plate with samples were put into the instruments with following procedures, enzyme activation at 95°C for 3 min, denaturation at 95°C for 15 s, annealing 95°C for 15 s, and dissociation by instruments, of which 40 cycle numbers was hold. The abundance of targeted bacteria was expressed by % of the total bacteria, which was calculated by the fold difference between the number of target gene copies and the number of 16S rRNA gene copies.

##### 2.3.4.3 Metagenomics Sequencing and Data Processing

The DNA sequencing libraries with insert of 350 bp were constructed following the manufacturer’s instruction (Illumina, San Diego, CA, USA). The libraries were then paired-end sequenced on the Illumina HiSeq high-throughput sequencing platform. The raw data were processed by MOCAT2 pipeline to remove low-quality reads, adapters, and human contamination, and then SOAP denova software were applied for assembling the clean data to obtain scaftigs. The taxonomic assignment and abundance estimation were conducted with metaphlan2 using default parameters. Subsequently, the comparisons of taxonomic between groups were conducted on statistical analysis of metagenomic profile (STAMP) software; for pathway analysis, CD-HIT software was used to get nonredundant genecatalogue (Unigenes) with the available effective scaftigs, then, DIAMOND software was applied to estimate the relative abundance of various functions (potential pathways) based on the Kyoto Encyclopedia of Gene and Genomes (KEGG) database. For the profiles in gene involved with carbohydrate enzymes, the referenced database is CAZy.

A pyrosequencing-based analysis of metagenomics was performed by using Illumina HiSeq platform to assess the regulating effects of oat on gut microbiota. The degrees of bacterial taxonomic similarity at species and genus levels were analyzed to assess the overall structure of the bacteria community between groups.

### 2.4 Statistical Analysis

Variables with data that had a normal distribution (including plasma lipids, ages, BMI, and SCFA) were expressed as mean ± SD values. Data from qPCR and metagenomics were expressed as relative abundance, and median and interquartile range values were used. Chi-square tests were used for categorical variables (gender) to examine the relationship between oat/rice group and gender. Independent-Samples *t*-test and paired-Samples *t*-test were employed to examine the significnce of plasma lipids and SCFAs between and within groups. Nonparametric Mann-Whitney *U*-test tests were performed to compare relative abundance of qPCR and White’s nonparametric *t*-test for metagenomic results and *p*-values were adjusted for multiple comparison using the false discovery rate (FDR). Pearson correlation was used to assess the relationship between blood lipids and SCFAs. Spearman correlation was conducted to examine the relationship between blood lipids and microbiota within groups. Correlation test was performed in SPSS (version 18.0, IBM, USA); others were run in R software with a 5% level of significance.

## 3 Results

### 3.1 Participant Demographic Information

There were 210 participants eligible for the study (70 in each site) and assigned equally into control and oat groups. During the study, 23 participants dropped out of which 11 participants were lost to follow-up (6 in control group and 5 in the oat group), with a loss to follow-up rate of 5%, and another 12 participants were excluded from the study, of which 8 did not take the samples as required (5 in control and 3 in oat group) and 4 decided not to continue the trial (1 in the control and 3 in the oat group). Therefore, final sample size was 187 participants, 93 in the control group and 94 in the oat group. There was no significant difference in general demographic characteristics between the groups at baseline (shown in **Table 1**).

**Table 1:** Demographic information of participants between control group and oat group at baseline (Day 0).

Characteristics	Control group (*n* = 93)	Oat group (*n* = 94)	*p-*value
Ages (years)	49.08** **±** **11.09	48.74** **±** **10.90	0.837
Gender (M/F)	64/29	65/29	0.961
BMI (kg/m^2^)	23.22** **±** **2.44	23.38** **±** **2.41	0.648

Data are expressed by mean ± SD. Independent-Samples t-test was used for ages and BMI. Chi-square test was used for gender.

A total of 180 and 177 samples were obtained from the two groups at baseline and endpoint for SCFA and metagenomic analysis, respectively. qPCR was performed only when sufficient fecal DNA was available following the metagenomics analysis. The exact number of samples used for qPCR, metagenomics, and plasma SCFA analysis are shown in **Supplementary Table S4**.

### 3.2 Blood Lipid Parameter Changes

**Figure 1:**
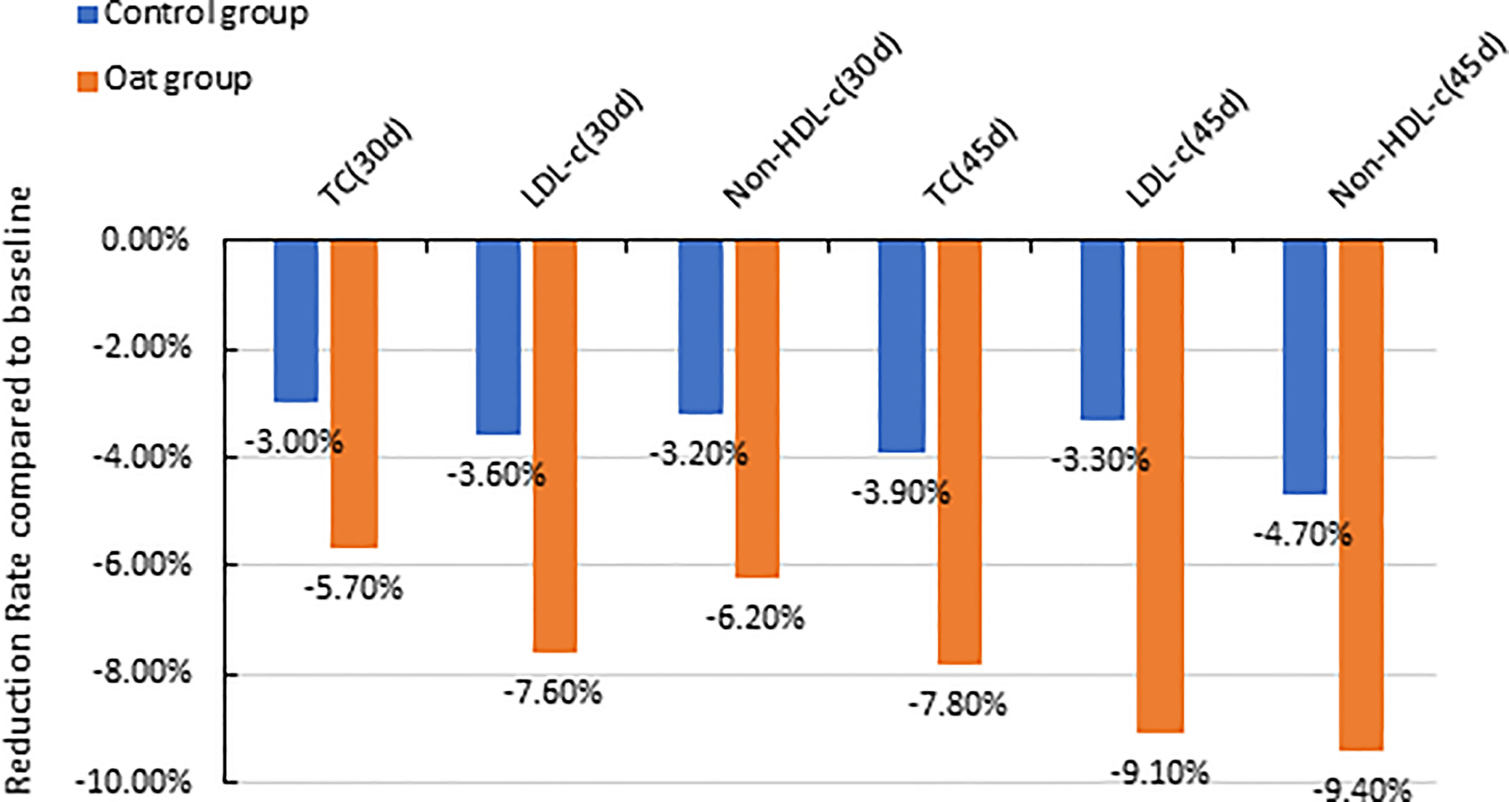
Barchart of percentage reduction of TC, LDL-c, and non-HDL-C at Day 30 and Day 45 compared with baseline (Day 0) for both oat (*n* = 94) and control (*n* = 93) groups (TC, total cholesterol; LDL-c, low-density lipoprotein cholesterol; non-HDL-C, Non-high-density lipoprotein cholesterol; d, Day).

The results showed that TC significantly decreased after 30-day intervention and after 45-day intervention in the oat group, compared with baseline (Day 0) (*p* < 0.001, *p* < 0.001, **Table 2**). The 5.7% and 8.7% decrease in TC were observed in oat groups at Days 30 and 45, respectively, compared with baseline (Day 0) (**Figure 1**). Significant decreases of TC, 3.0% and 3.9% at Days 30 and 45 respectively, were also observed in the control rice group (*p* = 0.002, *p* = 0.001). At Day 45, there was a significant difference in TC between the oat and control groups (*p* = 0.011).

**Table 2:** TC, TG, HDL-C, LDL-C, and non-HDL-C changes between groups and treatment periods.

	Control group (*n* = 93)	Oat group (*n* = 94)	*p*-value between groups
**TC (mmol/L)**			
Baseline (Day 0)	5.65** **±** **0.32	5.66** **±** **0.33	0.928
Day 30	5.48** **±** **0.56	5.34** **±** **0.57	0.113
Day 45	5.43** **±** **0.54	5.22** **±** **0.55	0.011
*p*-value within group (Day 0 vs. Day 30)	0.002	<0.001	
*p*-value within group (Day 0 vs. Day 45)	0.001	<0.001	
**TG (mmo/L)**			
Baseline (Day 0)	1.25** **±** **0.45	1.30** **±** **0.45	0.395
Day 30	1.37** **±** **0.54	1.36** **±** **0.64	0.972
Day 45	1.35** **±** **0.57	1.30** **±** **0.51	0.538
*p*-value within group (Day 0 vs. Day 30)	0.054	0.236	
*p*-value within group (Day 0 vs. Day 45)	0.101	0.808	
**LDL-C (mmol/L)**			
Baseline (Day 0)	3.32** **±** **0.45	3.41** **±** **0.41	0.154
Day 30	3.20** **±** **0.58	3.15** **±** **0.59	0.553
Day 45	3.21** **±** **0.56	3.10** **±** **0.50	0.166
*p*-value within group (Day 0 vs. Day 30)	0.072	<0.001	
*p*-value within group (Day 0 vs. Day 45)	0.083	<0.001	
**HDL-C (mmol/L)**			
Baseline (Day 0)	1.61** **±** **0.36	1.61** **±** **0.33	0.934
Day 30	1.57** **±** **0.37	1.55** **±** **0.35	0.798
Day 45	1.58** **±** **0.35	1.55 ± 0.33	0.558
*p*-value within group (Day 0 vs. Day 30)	0.104	0.082	
*p*-value within group (Day 0 vs. Day 45)	0.216	0.073	
**Non-HDL-C (mmol/L)**			
Baseline (Day 0)	4.04** **±** **0.42	4.05** **±** **0.37	0.885
Day 30	3.91** **±** **0.86	3.80** **±** **0.69	0.508
Day 45	3.85** **±** **0.75	3.67** **±** **0.76	0.049
*p*-value within group (Day 0 vs. Day 30)	0.006	<0.001	
*p-*value within group (Day 0 vs. Day 45)	0.001	<0.001	

Data are expressed by mean ± SD. Independent-Samples t-test was used for comparisons between groups. Paired-Samples t-test was used for comparisons within group.

There was a significant decrease in LDL-C after 30-day intervention and after 45-day intervention in oat group, compared with baseline (Day 0) (*p* < 0.001, *p* < 0.001, **Table 2**). In the oat group, a significant decrease of LDL-C of 7.6% after 30 days, and a decrease of 9.1% in LDL-C after 45-day intervention was observed (**Figure 1**).

### 3.3 Microbiota Changes (Both qPCR and Metagenomics)

Targeted microbiota enumeration by qPCR showed that, compared to baseline (Day 0), oat consumption significantly increased the abundance of *Akkermancia muciniphila* and *Roseburia* (*p* ****=** **0.04, *p* ****=** **0.02, respectively, shown in **Table 3**). There was a trend towards increased abundance of *Bifidobacterium* and *Faecalibacterium prausnitzii* in oat groups, though this was not statistically significant (*p* ****=** **0.51, *p* ****=** **0.32, respectively). A trend towards desrease in *Lactobacillus* population in both groups was observed (control: *p* ****=** **0.15; oat: *p* = 0.56, respectively). There was no difference before and after treatment or between treatment groups at either time point for the other bacteria enumerated.

**Table 3:** Abundance results from between (^#^) and within (*) group comparisons of 8 targeted bacterium.

	Control group	Oat group	*p*-value^#^
***Bifidobacterium* (%)**			
Baseline (Day 0)	0.29 (0.03, 1.08)	0.21 (0.03, 1.08)	0.52
Day 45	0.26 (0.03, 2.16)	0.42 (0.09,1.14)	0.62
*p* ^*^	0.70	0.51	
***Lactobacillus* (%)**			
Baseline (Day 0)	5.37 × 10^−4^ (1.06 × 10^−4^, 7.84 × 10^−3^)	3.80 × 10^−4^ (3.99 × 10^−5^, 3.70 × 10^−3^)	0.43
Day 45	2.49 × 10^−4^ (2.20 × 10^−5^, 1.30 × 10^−3^)	1.90 × 10^−4^ (3.5 × 10^−6^, 4.62 × 10^−3^)	0.84
*p* ^*^	0.15	0.56	
***Akkermancia muciniphila* (%)**			
Baseline (Day 0)	2.04 × 10^−3^ (2.1 × 10^−6^, 3.28 × 10^−1^)	1.74 × 10^−3^ (0, 2.03 × 10^−1^)	0.85
Day 45	7.09 × 10^−5^ (0, 4.93 × 10^−2^)	2.84 × 10^−3^ (1.34 × 10^−5^, 1.47 × 10^−1^)	0.06
*p* ^*^	0.09	0.04	
***Roseburia* (%)**			
Baseline (Day 0)	10.71 (3.83, 20.24) n=36)	2.05 (0.58, 11.34)	0.001
Day 45	8.07 (2.96, 20.95) n=31)	5.87 (2.70, 22.49)	0.73
*p* ^*^	0.51	0.02	
***Bacteroidaceae* (%)**			
Baseline (Day 0)	1.05 (0.42, 2.84)	0.85 (0.16, 2.37)	0.37
Day 45	1.02 (0.34, 3.83)	0.90 (0.28, 2.44)	0.67
*p* ^*^	0.87	0.84	
***Faecalibacterium prausnitzii* (%)**			
Baseline (Day 0)	0.67 (0.09, 1.49)	0.40 (0.04, 1.74)	0.49
Day 45	0.19 (0.04, 2.53)	0.83 (0.08, 4.16)	0.43
*p* ^*^	0.79	0.32	
***Enterobacteriaceae* (%)**			
Baseline (Day 0)	5.18 × 10^−3^ (2.82 × 10^−4^, 1.20 × 10^−1^)	4.53 × 10^−3^ (5.96 × 10^−4^, 6.96 × 10^−2^)	0.85
Day 45	3.10 × 10^−3^ (1.22 × 10^−4^, 2.84 × 10^−2^)	4.35 × 10^−3^ (1.74 × 10^−4^, 2.39 × 10^−2^)	0.84
*p* ^*^	0.32	0.42	
***Clostridium perfringens (samples from Shanghai were not be detected)* (%)**			
Baseline (Day 0)	1.28 × 10^−4^ (7.3 × 10^−6^, 9.36 × 10^−4^)	3.00 × 10^−4^ (1.16 × 10^−5^, 6.07 × 10^−3^)	0.46
Day 45	3.49 × 10^−3^ (9.3 × 10^−6^, 9.52 × 10^−2^)	5.56 × 10^−4^ (1.33 × 10^−5^, 3.00 × 10^−2^)	0.61
*p* ^*^	0.14	0.61	

Data were presented as median (P25, P75). Nonparametric Mann-Whitney U test was used for comparisons between and within groups.

### 3.4 Microbiota Changes by Using Metagenomics

**Figure 2:**
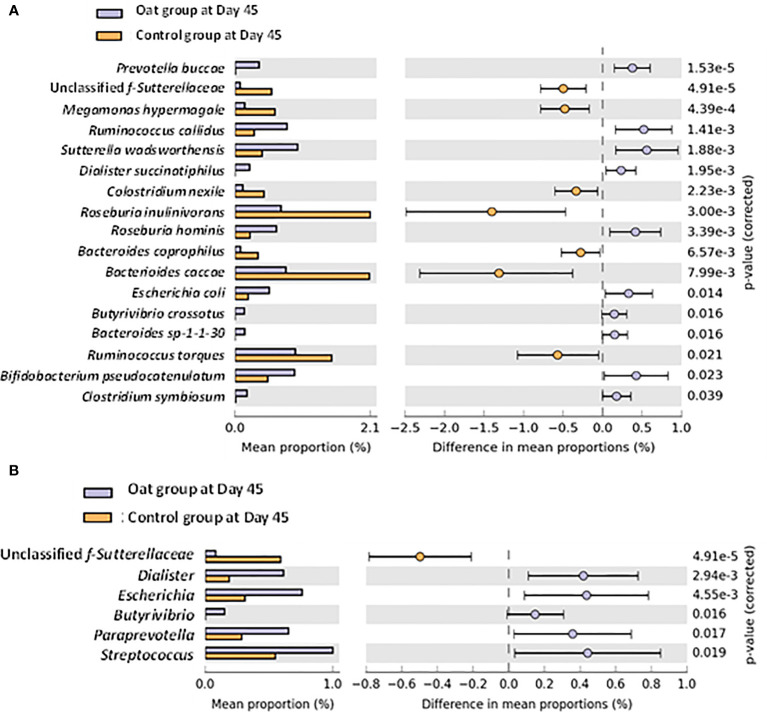
**(A)** Barchart of significant changes of bacterium at species level between oat and control groups after 45-day intervention (White’s nonparametric *t*-test after FDR was used for comparison between groups; analysis was performed on STAMP software). **(B)** Barchart of significant changes of bacterium at genus level between oat and control groups after 45-day intervention (White’s nonparametric *t*-test after FDR was used for comparison between groups; analysis was performed on STAMP software).

A total of 450 of bacteria were identified by using shotgun metagenomic within both groups. No significant differences were observed for microbial diversity indices, including alpha and beta diversities (**Supplementary Figures S1–5**). However, significant differences in specific bacteria at species and genus level were observed after intervention. Univariate analysis at species level showed that the relative abundance of *Prevotella buccae*, *Dialister succinatiphilus*, *Roseburia hominis*, *Butyrivibrio crossotus*, *Bifidobacterium pseudocatenulatum*, and *Clostridium symbiosum* increased significantly in the oat group compared with the control group after Day 45 interventions (*p* < 0.05), while unclassified f-*Sutterellaceae*, *Megamonas hypermegale*, *Clostridium nexile*, and *Roseburia inulinivorans* showed a notable decrease (**Figure 2A**). In addition, at the genus level, oat consumption significantly increased the relative abundance of *Dialister*, *Butyrivibrio*, and *Paraprevotella* and decreased unclassified *f-Sutterellaceae* compared with the control group (**Figure 2B**). These findings indicated that oat consumption induced significant shifts in specific members of the gut microbiota.

**Figure 3:**
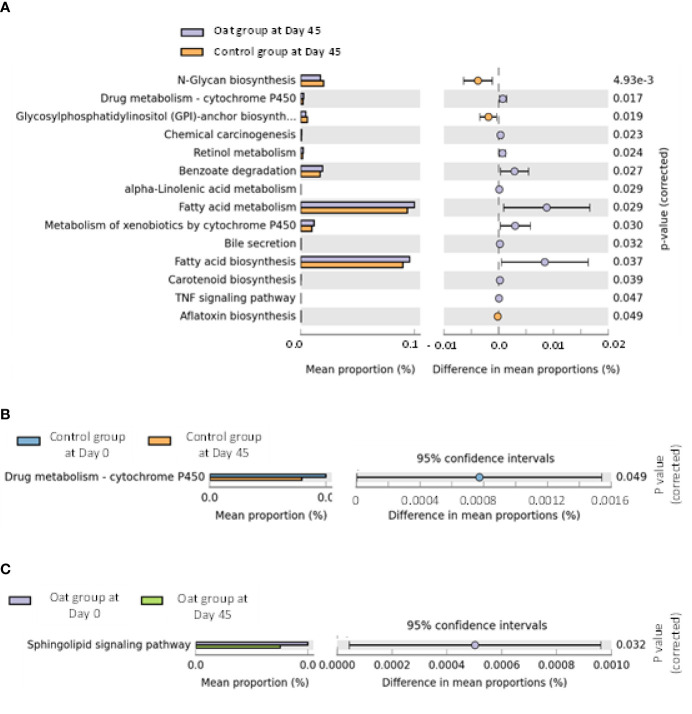
**(A)** Barchart of metabolic pathways which are significantly related to oat consumption between oat and control groups (White’s nonparametric *t*-test after FDR was used for comparison between groups; analysis was performed on STAMP software and referenced to KEGG data). **(B)** Barchart of significantly different pathways within control group at days 0 and 45 in two sites (White’s nonparametric *t*-test after FDR was used for comparison within group; analysis was performed on STAMP software and referenced to KEGG data). **(C)** Barchart of significantly different pathways within oat group at days 0 and 45 in two sites (White’s nonparametric *t*-test after FDR was used for comparison within group; analysis was performed on STAMP software and referenced to KEGG data).

The pathway analysis showed that oat consumption for 45 days induced significant differences in fatty acid metabolism and fatty acid biosynthesis, and other metabolic pathways (shown in **Figures 3A–C**).

**Figure 4:**
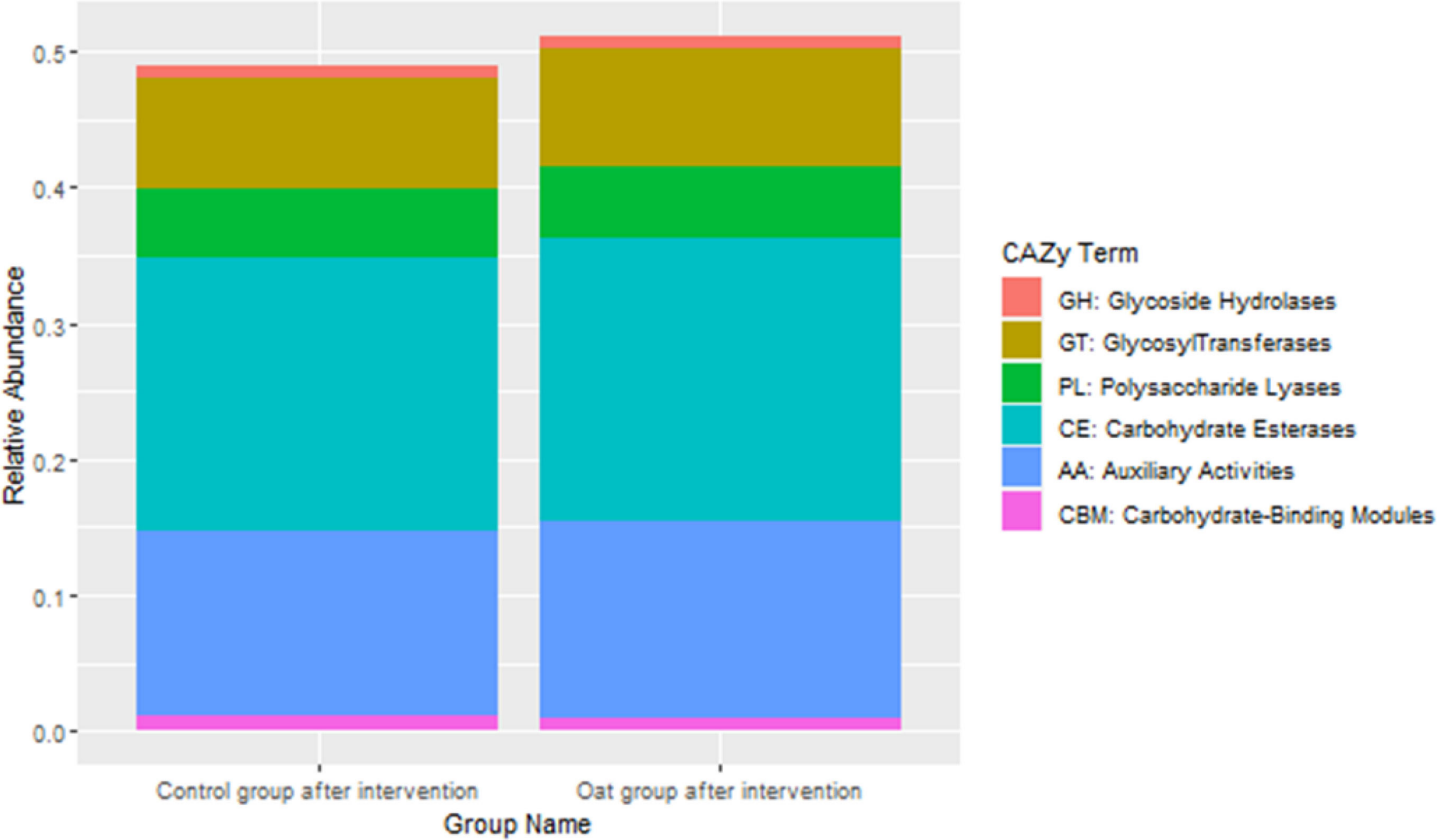
Barchart of distributions of various carbohydrate enzymes based on CAZy database between groups after interventions.

CAZy database suggested that after oat intervention, there were some changes in profiles of various carbohydrate enzymes, including increased carbohydrate esterases and glycosyltransferases, which is shown in **Figure 4**.

### 3.5 Relationship Between Microbiota and Blood Lipid Parameters

The correlation results showed that, in oat group, *Bifidobacterium* was negatively correlated to LDL-C (*p* = 0.01, *r* = −0.31). *Lactobacillus* was positively correlated to LDL-C (*p* = 0.03, *r* = 0.29).

**Figure 5:**
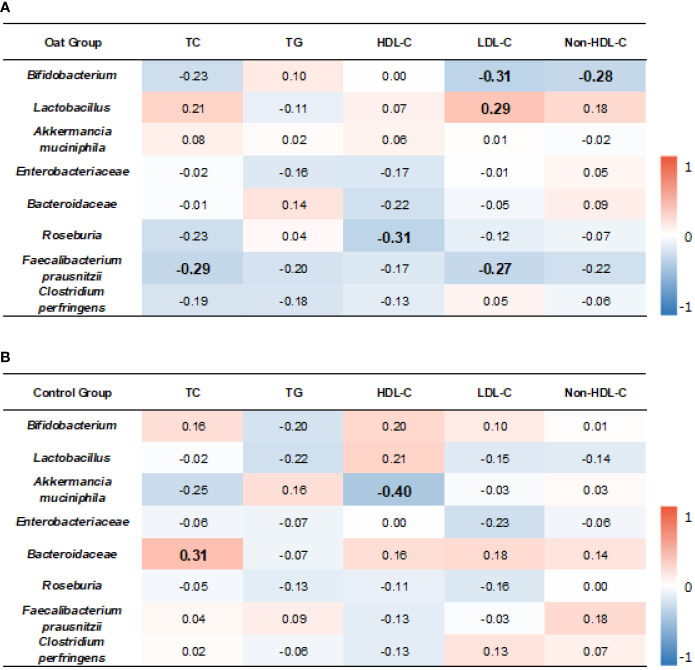
Heatmap of correlation coefficients between bacterium and blood lipid parameters in oat group **(A)** and in control **(B)** group. Correlation values in bold indicate significance. TC, total cholesterol; TG, total triglyceride; HDL-C, high-density lipoprotein cholesterol; LDL, low-density lipoprotein cholesterol; non-HDL-C, non-high-density lipoprotein cholesterol. Correlation analysis was based on Spearman correlation method.

TC and LDL-C were negatively correlated to *Faecalibacterium prausnitzii* (*p* = 0.02, *r* = −0.29; *p* = 0.03, *r* = −0.27, respectively). HDL-C was negatively correlated to *Roseburia* (*p* = 0.01, *r* = −0.31) (**Figure 5A** **).**

In the control group, *Akkermancia muciniphila* was negatively correlated to HDL-C (*p* = 0.006, *r* = −0.40) and *Bacteroidaceae* was positively correlated to TC (*p* = 0.01, *r* = 0.31) (**Figure 5B** **).**

### 3.6 SCFA Changes

Compared with Day 0, oat consumption for 45 days significantly increased plasma acetic acid (*p* = 0.03) and propionic acid (*p* = 0.05); of note, a similar increase was also observed in control group (*p* = 0.01 for acetic acid and *p* = 0.009 for propionic acid respectively). No significant effect of oat consumption was found in other SCFAs. Furthermore, for all SCFAs determined, similar change pattern of SCFAs were found in the two groups. **Table 4** shows the detailed changes of plasma SCFAs between and within groups over the course of the trial.

**Table 4:** Plasma SCFAs concentrations (mg/L) between (^#^) and within (*) groups comparisons.

	Control group	Oat group	*p* ^#^		Control group	Oat group	*p* ^#^
**Acetic acid** (mg/L)				**Valeric acid** (mg/L)			
**Baseline (Day 0)**	3.84** **±** **0.79	3.92** **±** **0.85	0.64	**Baseline (Day 0)**	0.17** **±** **0.02	0.17** **±** **0.02	0.57
**Day 45**	4.37** **±** **1.08	4.37** **±** **1.10	0.98	**Day 45**	0.17** **±** **0.03	0.17** **±** **0.02	0.57
*p* ^*^	0.01	0.03		*P* ^*^	0.34	0.17	
**Propionic acid** (mg/L)				**Isovaleric acid** (mg/L)			
**Baseline (Day 0)**	0.33** **±** **0.11	0.35** **±** **0.08	0.44	**Baseline (Day 0)**	2.49** **±** **0.59	2.64** **±** **0.63	0.24
**Day 45**	0.40** **±** **0.12	0.39** **±** **0.12	0.69	**Day 45**	2.63** **±** **0.58	2.64** **±** **0.44	0.94
*p* ^*^	0.009	0.050		*P* ^*^	0.29	0.96	
**Butyric acid** (mg/L)				**Hexanoic acid** (mg/L)			
**Baseline (Day 0)**	0.86** **±** **0.82	0.96** **±** **0.82	0.56	**Baseline (Day 0)**	0.32** **±** **0.05	0.32** **±** **0.04	0.81
**Day 45**	1.00** **±** **1.02	1.03** **±** **0.95	0.9	**Day 45**	0.31** **±** **0.05	0.31** **±** **0.05	0.99
*p* ^*^	0.45	0.68		*p* ^*^	0.51	0.64	
**Isobutyric acid** (mg/L)							
**Baseline (Day 0)**	1.08** **±** **0.30	1.13** **±** **0.28	0.35				
**Day 45**	1.04** **±** **0.30	1.12** **±** **0.28	0.18				
*p* ^*^	0.56	0.86					

Data are expressed by mean ± SD. Independent-Samples t-test was used for comparisons between groups. Paired-samples t-test was used for comparisons within group.

### 3.7 Relationship Between Microbiota Changes and SCFA Changes

**Figure 6:**
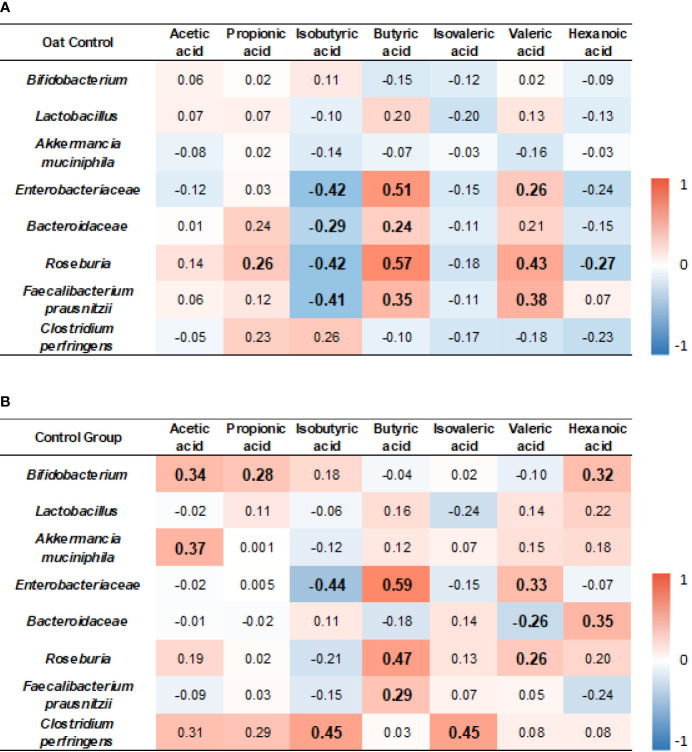
Heatmap of correlation coefficients between bacterium and SCFAs in oat group **(A)** and in control **(B)** group. Correlation values in bold indicate significance. TC, total cholesterol; TG, total triglyceride; HDL-c, high-density lipoprotein cholesterol; LDL, low-density lipoprotein cholesterol; non-HDL-c, non-high-density lipoprotein cholesterol. Correlation analysis was based on Spearman correlation method.

The results showed that, in the oat group, *Enterobacteriaceae* was positively correlated to butyric acid and valeric acid (*p* < 0.001, *r* = 0.51; *p* = 0.045, *r* = 0.26, respectively), but negatively correlated to isobutyric acid (*p* = 0.001, *r* = −0.42). *Roseburia* was positively correlated to propionic acid, butyric acid, and valeric acid (*p* = 0.04, *r* = 0.26; *p* < 0.001, *r* = 0.57; *p* < 0.001, *r* = 0.43, respectively), but negatively correlated to isobutyric acid and hexenoic acid (*p* = 0.01, *r* = −0.42; *p* = 0.04, *r* = −0.27, respectively). *Faecalibacterium prausnitzii* was negatively correlated to isobutyric acid (*p* = 0.001, *r* = −0.41) but positively correlated to butyric acid and valeric acid (*p* = 0.005, *r* = 0.35; *p* = 0.002, *r* = 0.38, respectively). The detailed correlation coefficients are shown in **Figure 6A**.

In the control group, *Bifidobacterium* was positively correlated to acetic acid, propionic acid, and hexanoic acid (*p* = 0.01, *r* = 0.34; *p* = 0.03, *r* = 0.28; *p* = 0.02, *r* = 0.32, respectively). *Akkermancia muciniphila* was positively correlated to acetic acid (*p* = 0.02, *r* = 0.37). *Enterobacteriaceae* was positively correlated to butyric acid and valeric acid (*p* < 0.001, *r* = 0.59; *p* = 0.01, *r* = 0.33, respectively), but negatively correlated to isobutyric acid (*p* = 0.001, *r* = −0.44). *Roseburia* was positively correlated to butyric acid and valeric acid (*p* < 0.001, *r* = 0.41; *p* < 0.001, *r* = 0.57; *p* = 0.04, *r* = 0.26, respectively). *F. prausnitzii* was positively correlated to butyric acid (*p* = 0.03, *r* = 0.29, respectively). *Clostridium perfringens* was positively correlated to isobutyric acid and isovaleric acid (*p* = 0.02, *r* = 0.45; *p* = 0.03, *r* = 0.45, respectively). The detailed correlation coefficients are shown in **Figure 6B**.

### 3.8 Relationship Between SCFA Changes and Blood Lipid Parameters

The results showed that, in all the participants, isobutryric acid was positively correlated to LDL-C (*r* = 0.21, *p* = 0.006). In addition, the isovaleric acid was positively correlated to TG (*r* = 0.25, *p* = 0.001) and non-HDL-C (*r* = 0.20, *p* = 0.012). The HDL-C was negatively correlated to butyric acid (*r* = −0.20, *p* = 0.009), isovaleric acid (*r* = −0.23, *p* = 0.003), and valeric acid (*r* = −0.17, *p* = =0.029).

**Figure 7:**
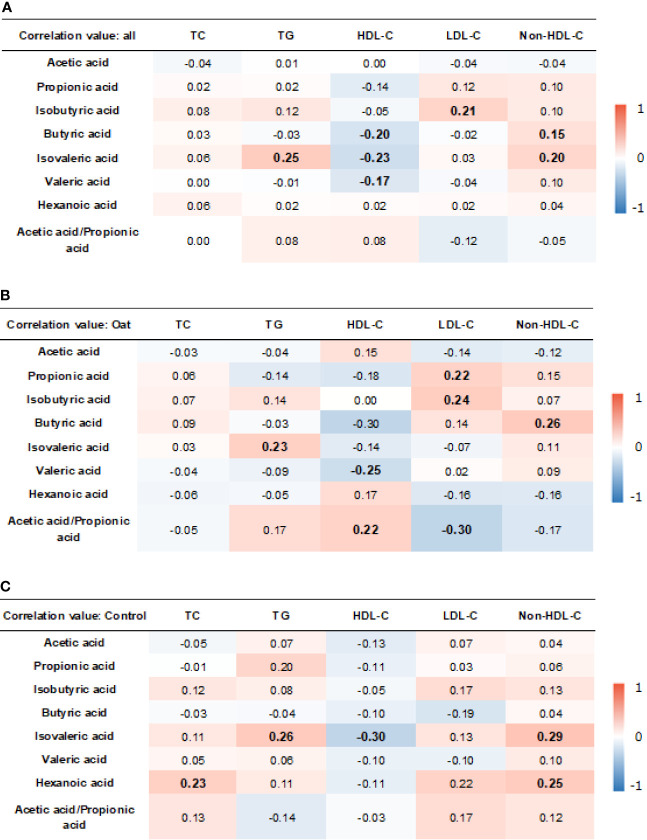
Heatmap of correlation coefficients between blood lipid parameters and SCFAs in all participants **(A)**, oat group **(B)**, and in control **(C)** group. Correlation values in bold indicate significance. TC, total cholesterol; TG, total triglyceride; HDL-c, high-density lipoprotein cholesterol; LDL, low-density lipoprotein cholesterol; non-HDL-c, non-high-density lipoprotein cholesterol. Correlation analysis was based on Pearson correlation method.

In the oat group, HDL-C was negatively correlated to valeric acid (*p* = 0.02, *r* = −0.25). TG was positively correlated to isovaleric acid (*p* = 0.03, *r* = 0.23) in the oat group. In addition, a positive correlation was found in the oat group between LDL-C and propionic acid (*p* = 0.049, *r* = 0.22) and between LDL-C and isobutyric acid (*p* = 0.02, *r* = 0.24). There were significant negative relationships between the acetate:propionate ratio and LDL-C (*r* = −0.30, *p* = 0.005). The detailed correlation coefficients in all participants and each group are shown in **Figures 7A–C**, respectively.

## 4 Discussion

In the current study, we demonstrated that consuming 80 g of oats, containing 3.0 g of β-glucan and 56.8 mg polyphenol, for 45 days could effectively reduce TC and LDL-C in hypercholesterolemic Chinese subjects. Moreover, we demonstrated that oat consumption significantly increased the abundance of bacteria previously shown to protect against metabolic disease, obesity, and CHD, specifically, *Akkermansia mucinophila* and *Roseburia*, as well as other saccharolytic and butyrate producing members of the gut microbiota. This remodeling of the microbiome resulted in a significant increased relative abundance of genes involved in microbiome fatty acid biosynthesis and fatty acid metabolism. We also showed that oats intake significantly increased fasting plasma concentrations of acetate and propionate, providing a putative mechanistic link between oat-induced microbiota modulation and blood cholesterol homeostasis. Although the control group showed that a reduction in TC is also concomitant with increased plasma acetate and propionate concentrations, oat consumption resulted in greater reduction (7.8%) in TC compared with the control group (3.9%). Our finding that oat consumption lowered cholesterol was consistent with previous studies (22, 26, 27), as well as the conclusion of meta-analyses, that also showed the consumption of oats and oat-derived β-glucan can effectively lower TC and LDL-C (5).

Both animal studies and human clinical studies have explored the influence of oat β-glucan on the gut microbiota, most of which have shown that the consumption of oats and oat β-glucan could significantly increase the abundance of *Bifidobacterium* and *Lactobacillus* (22, 28–30). Kristek et al. using an *in vitro* model of human microbiota fermentation found that oat bran had a greater impact on microbiota composition, increasing *bifidobacteria* as well as acetate and propionate productions, than individual bioactive components oat β-glucan or oat polyphenols (31). Similarly, oat bran (30 g/day), containing 8.9 g/day dietary fiber, has recently shown to reduce blood pressure and increase fecal *bifidobacteria* in a Chinese population (32). However, we did not find a statistically significant increase in *bifidobacteria* and *lactobacilli* after oat consumption, although a trend was apparent in qPCR data for *bifidobacteria* and *F. prausnitzii*. Since the gut microbiota are easily affected by dietary patterns (33), we speculated that the reasons behind may be related to a small sample size which were collected from both the Nanjing and Shanghai sites.

*A. muciniphila* have been reported to play an important role in metabolic disease (34, 35). In the present study, we did observe that oat consumption significantly increased *A. muciniphila* and *Roseburia*, which showed a high consistency with previous studies. Ryan et al. found that oat β-glucan increases the abundance of *A. muciniphila* (36). Moreover, Depommier et al. showed that probiotic supplementation using *A. muciniphila* was inversely related to TC, in a randomized double-blind, placebo-controlled pilot study of 32 overweight/obese insulin-resistant volunteers (34). In addition, Mitsou et al. showed that colonization patterns of *A. muciniphila* in a Greek adult population were associated with cardiometabolic markers and adiposity (37). In addition, in metagenomic results, we found that oat consumption could significantly increase the relative abundance of *Dialister*, *Butyrivibrio*, and *Paraprevotella* and decreased unclassified *f-sutterellaceae* at the genus level, showing some similarities to other dietary interventions rich in fiber and polyphenols, in which the authors all reported a reduction of TC and LDL-C upon the dietary interventions in healthy subjects and rodent models of metabolic disease (38, 39). *Roseburia* has also shown the link to improve the cardiometabolic profiles and a main butyrate producer within the gut microbiota; it is reported that there is a negative relationship between *Roseburia* and TC and LDL-C (40). In our present study, *Roseburia* and *F. prausnitzii*, another major butyrate producers, were positively correlated with plasma butyrate concentrations. These results also provided the evidences for the beneficial effects of SCFAs on human metabolism (19). Although our results failed to find significant relationships between *A. muciniphila* and TC and LDL-C, neither between *Roseburia* and TC and LDL-C, our results did indicate that in the oat group, *Bifidobacterium* and *F. prausnitzii* were negatively correlated to LDL-C. In addition, *F. prausnitzii* was also negatively correlated to TC. Moreover, our pathway analysis of the metagenomics dataset revealed significant increased abundance of genes involved in fatty acid biosynthesis and fatty acid metabolism within the gut microbiota after ingestion of oats. Interestingly, the gut microbiota has been shown to modulate fatty acid profiles in plasma, liver, and the intestine (41, 42), which is in accordance with the metabolomics analysis from our published study; we found that oats induced specific changes in fatty acid within the human metabolome, specifically a reduction in glycerophospholipid and sphingolipids (23). Such observations call for further studies examining the contribution of the gut microbiota to the mammalian lipidome and its role in regulating host energy and lipid metabolism.

The current study focused on oat β-glucan; however, the effect of polyphonels on gut microbita and health benefits cannot be ingored. As a good source of phytochemicals, oats contain a number of phenolic acids which could serve as a complex molecule by combining with soluble esters, proteins, and other macromolecules, such as ferric acid and vanillic acid (43, 44). Of note, there is an another unique molecular weight-soluble phenolic compounds for oats, the avenanthramides (AVAs), and they were first purified from oat groats and hulls by Collins, and mainly existed in the oat bran and aleurone layer (45). The predominant AVAs found in oats are 2c, 2f, and 2p according to the systematic nomenclature developed by Dimberg (46). The antioxidant properties of AVAs have been verified in numberous clinical trials (47). In addition, in terms of lipids metabolism, AVAs showed a cholesterol-lowering property by notably decreasing the level of TC, TG, and LDL-C in healthy subjects (48). As an important phytochemicals, oat polyphenols could also improve host health by interacting with intestinal immune system and, in some cases, the gut microbiota. Reviews by Angelika et al. have listed the detailed actions between polyphenol intake and immune system, including modulation of T-cell functions and downregulation of inflammtiry cytokine responses (49). For AVAs, previous *in vitro* studies suggested that oat AVAs have an anti-atherosclerosis effect *via* inhibition of adhesion molecule expression and proinflammatory cytokines and chemokines (50); also, the inhibition of vascular smooth muscle cell proliferation and stimulation of NO production may also participate in this effect (51). In clinical trial, Liu et al. found that supplementation of oat AVAs with 3.12 mg daily for 1 month can significantly reduce the level of TC and LDL-C by 11.1% and 15.1%, respectively (48). However, Kristek et al. claimed that the greatest impact on gut microbiota could appear only when oats as a whole food, rather than its main bioactives β-glucan or polyphenols alone (31). These results showed a high accordance with the results of the present study.

The International Scientific Association for Probiotics and Prebiotics defined a prebiotic as “a substrate that is selectively utilized by host microorganisms conferring a health benefit” (52). According to this definition, a prebiotic should be selectively utilized by host microorganisms, preferably beneficial members of the gut microbiota and also confer a health benefit on the host. Health benefits of oats and oat-derived products containing at least 3.0 g β-glucan are well established (53) and confirmed in this study among Chinese population. We also report here a specific modulation of the gut microbiota upon oat ingestion, leading to increased abundance of bacteria associated with improved metabolic health, specifically *Akkermansia muciniphila* and *Roseburia*, and with a trend towards increased abundance of *Bifidobacterium* and *Faecalibacterium prausnitzii* and increased relative abundance of saccharolytic and butyrate-producing members of the gut microbiota upon metagenomics analysis, all of which has been shown to respond to dietary interventions of lowering TC and LDL-C. Importantly, these microbiota-induced changes were restricted to a limited number of bacterial taxa and the effect was not observed in the rice group. This selective microbiota modulation is consistent with the few previous studies examining the impact of oats and β-glucans on the gut microbiota (20, 22, 28, 30).

SCFAs produced from fiber or prebiotic fermentation by the gut microbiota have been shown in preclinical settings and in small human mechanistic studies to not only regulate TC and LDL-C but also to regulate food intake and influence fat storage in adipose tissue, thermogenesis, and browning of adipose tissue, all of which influence cholesterol homeostasis (54–57). Although acetate is a substrate for hepatic cholesterol synthesis, propionate inhibits acetate utilization for cholesterol synthesis in humans (58). Indeed, the ratio of serum acetate:propionate has been shown to be positively associated with total cholesterol levels, at least in men (59). Similarly, circulating SCFAs, particularly acetate and propionate, have been associated with peripheral insulin sensitivity, whole body lipolysis, and glucagon-like peptide-1 (GLP-1) concentrations (60), although possible sex effects may play a confounding role (61). GLP-1 influences lipid metabolism *via* lipoproteins (62), and the influence of SCFAs and BAs on whole body lipolysis, adipose tissue metabolism, thermogenesis, and insulin sensitivity identifies the gut microbiota and diet-induced modulation of gut microbiota metabolic output as plausible regulators of cholesterol homeostasis and CHD risk.

The *in vitro* study conducted by Kim and White found that, by adding oat flake into the fermentation model, oat flake could significantly increase the productions of SCFAs, including acetic acid, propionic acid, and butyric acid (63). Connolly et al. did not find statistically significant differences in SCFA changes between the oat and control group (20). Velikonja et al. found that subjects consuming 6 g of barley β-glucan bread showed a significant increase in propionic acid (22). The influence of β-glucan on specific SCFA changes is not highly consistent. In the present trial, we did observed significant increases of acetic acid and propionic acid in both groups. One reason could be due to the fecal samples used in literature whereas plasma samples used in present study for SCFAs analysis. According to Borthakur et al., the use of fecal SCFAs might not accurately reflect the colonic SCFA production from fermentation (64), because SCFAs can induce their own active uptake transporter on intestinal epithelial wall. Therefore, both fecal samples and plasma samples are suggested to collected for SCFA analysis in the future study, in order to obtain a better understating on the influences of β-glucan on SCFA changes.

In animal studies, whole grain oat intake has been found to increase valeric acid production, and *Bifidobacterium*, *Lactobacillus*, and butyrate-producing bacteria including *Roseburia*. Valeric acid production in pigs was correlated with *bifidobacteria* and *lactobacilli* (65). In the current trial, we found a negative correlation between HDL-C and valeric acid in the oat group, which caused some discrepancies with the results of mechanistic studies performed in male Syrian hamsters, that ingestion of valeric acid did not change TC but did reduce non-HDL-C and improved the ratio of non-HDL-C to HDL-C (66). We speculated that the different absorptions and metabolic patterns of valeric acid in different species may contribute to the present inconsistent results and need to be further studied. On the other hand, butyric acid and valeric acid were found to be positively correlated to *Enterobacteriaceae*, *Roseburia*, and *Faecalibacterium prausnitzii* in the oat group, all these three bacteria were negatively correlated to isobutyric acid. Consistently, the study performed by Lu et al. showed that treatment in obese mice with a mixture of butyrate has been proven to improve the plasma lipid profile *via* G protein-coupled receptors. In fact, butyric acid is the preferred energy source for colonocytes and can inhibit isobutyrate catabolism by competitively inhibiting activation of isobutyrate to its CoA ester; whereas, when colonocytes express a low butyrate availability, isobutyrate can function as a carbon source for energy (67). In other words, butyrate may present an opposite role to isobutyrate. Of note, we also observed a positive correlation in the oat group between LDL-C and isobutyric acid, which indicated the beneficial effects of butyrate indirectly and made us more interested to explore how the oat consumption improve lipid profiles through the potential metabolites of SCFAs.

SCFA production therefore, may represent a possible mechanism by which diet-induced microbiota modulation could contribute to the cholesterol-lowering effect of oats. The study of Anderson et al. found that, the cholesterol synthesis was inhibited by 1–1.25 mmol/L propionic acid (68). Moreover, Wolever et al. and Wong et al. speculated that SCFAs could inhibit the synthesis of 3-hydroxy-3-methylglutaryl coenzyme A synthetase and reductase to inhibit cholesterol synthesis (58) (69). In conclusion, oat consumption containing 3.0 g β-glucan and 56.8 mg polyphenol effectively reduced TC and LDL-C and induced a notable alteration in intestinal microbiota structure. *Akkermansia muciniphila*, *Roseburia*, *Bifidobacterium*, and *Faecalibacterium prausnitzii* can act as critical roles in lowering cholesterols after oat consumption, as well as the production of valeric acid.

There are some limitations of the current study which should be considered. First of all, the control treatment, 80 g/day rice for 45 days, also induced significant changes in blood lipid profiles, although not to the same extent as oats. The significantly reduced nutrients intake in control group compared with baseline may provide some explanation for this phenomenon (**Supplementary Table 5**). In addition, since the participants were subjects with dyslipidemia, they are eager to keep healthy; in this way, some effects related to placebo or expectancy may cause some biases if volunteers paid more attention to their lifestyles after participating in the trial. On the other hand, it was important for us to use real foods in this experiment. The cholesterol-lowering effects of 3 g oat β-glucan are well established, but the ability of oats, as a whole food to modulate the gut microbiota and their metabolic output is poorly studied. We also chose rice as a control to help us confirm the prebiotic nature of oats, modulating the gut microbiota and mediating a health effect compared with an equivalent, nonprebiotic cereal. We believe that this real-world situation provides a stronger demonstration both of the benefit of oats in normalizing blood lipid profiles in hypercholesterolemic subjects and in mediating a prebiotic modulation of the gut microbiota. Another limitation is that, the relationships between blood lipids, gut microbiota, and SCFAs were concluded from the statistical method of correlation analysis only, which may not be reliable but offer new clues for our next experiments aiming to explore the causal relationships through fecal microbiota transplantation technology. Lastly, we were not able to obtain the fecal samples from the participants from the Beijing site, resulting in a relatively small sample size for metagenomics analysis. Larger sample size with well-designed trials is required to obtain further evidence.

## 5 Conclusion

In summary, our study demonstrated that oats exhibited prebiotic activity and ability of oats to modulate microbiota showed a preliminary causal relationship with its cholesterol-lowering ability in mild hypercholesterolemic individuals.

## Data Availability Statement

The raw metagenommics sequence data were deposited into the National Microbiology Data Center (https://nmdc.cn), and the accession number is NMDC40012570; other data that support the findings of this study are available from the corresponding author upon reasonable request.

## Ethics Statement

The studies involving human participants were reviewed and approved by the Chinese Clinical Trial Registry (www.chictr.org.cn) and was given a favorable ethics evaluation and approved by the China Ethics Committee of Registering Clinical Trials (ChiECRCT-20180139). The patients/participants provided their written informed consent to participate in this study.

## Author Contributions

Conception and design of the study: GS, YY, and DX. Experiment execution: DX, SW, DP, HL and JS. Collection of data: SW, DP, HL and JS. Analysis and interpretation of data: MF, YC, FL, XY, CY, BZ, NL, XW, QX. Drafting of the manuscript: DX, YC, XZ, KT, VS. Critical revision of the manuscript: MF, VS, KT, AK. Administrative support and study supervision: GS, YY and JS. All authors contributed to the article and approved the submitted version.

## Funding

The project was funded by the Chinese Nutrition Society and PepsiCo, Inc., China. The founders play no role in the conduction of the trial. The funds received for open access publication fees are from our institution.

## Author Disclaimer

The views expressed in this article are those of the authors and do not necessarily reflect the opinion or policies of PepsiCo, Inc.

## Conflict of Interest

Authors MF, YC, VS, FL, XZ and AK were employed by company PepsiCo, Inc.

The remaining authors declare that the research was conducted in the absence of any commercial or financial relationships that could be construed as a potential conflict of interest.

## Publisher’s Note

All claims expressed in this article are solely those of the authors and do not necessarily represent those of their affiliated organizations, or those of the publisher, the editors and the reviewers. Any product that may be evaluated in this article, or claim that may be made by its manufacturer, is not guaranteed or endorsed by the publisher.
