# Supplementary material for: The Prebiotic Effects of Oats on Blood Lipids, Gut Microbiota, and Short-Chain Fatty Acids in Mildly Hypercholesterolemic Subjects Compared With Rice: A Randomized, Controlled Trial
Source: Front Immunol. 2021 Dec 9;12:787797. doi: 10.3389/fimmu.2021.787797 (PMC8697019; doi:10.3389/fimmu.2021.787797)
Supplement: Supplementary file 1 [file DataSheet_1.docx]

**Supplementary files**

**Table 1** Nutrient composition of tested products

| Nutrients | | | Oats | Rice |
| --- | --- | --- | --- | --- |
| Energy (kcal) | | | 304 | 311 |
| Protein (g) | | | 8.8 | 5.3 |
| Fat (g) | | | 7.4 | 1.5 |
| Carbohydrate(g) | Total | | 48.4 | 67.7 |
|  | Fiber | | 9.6 | 0.7 |
|  | β-glucan | | 3.0 | 0.0 |
| Polyphenol | Total polyphenol (mg) | | 56.8 | 0.0 |
|  | Avenanthramides | 2c (µg) | 104 | 0.0 |
|  |  | 2p (µg) | 102.4 | 0.0 |
|  |  | 2f (µg) | 151.5 | 0.0 |

**Table 2** Primers and TaqMan probes targeting 16S rRNA coding regions of bacteria for targeted 8 bacteria

| Target bacterium | Primer/probe | Sequence (5’-3’) | Reference |
| --- | --- | --- | --- |
| *Bifidobacterium spp.* | F | GCGTGCTTAACACATGCAAGTC | [1] |
|  | R | CACCCGTTTCCAGGAGCTATT |  |
|  | Probe | FAM-TCACGCATTACTCACCCGTTCGCC-TAMRA |  |
| *Lactobacillus spp* | F | TGGATGCCTTGGCACTAGGA | [2] |
|  | R | AAATCTCCGGATCAAAGCTTACTTAT |  |
|  | Probe | FAM-TATTAGTTCCGTCCTTCATC-TAMRA |  |
| *Akkermansia Muciniphila* | F | CAGCACGTGAAGGTGGGGAC | [3] |
|  | R | CCTTGCGGTTGGCTTCAGAT |  |
|  | Probe | FAM-CCCCACCTTCCTCCCAGTTGAT-TAMRA |  |
| *Faecalibacterium prausnitzii* | F | CCCGGCATCGGGTAGAG | [4] |
|  | R | GGACGCGAGGCCATCTC |  |
|  | Probe | FAM-AAAAGGAGCAATCCGCT-TAMRA |  |
| *Enterobacteriaceae* | F | GCGGTAGCACAGAGAGCTT | [5] |
|  | R | GGCAGTTTCCCAGACATTACTCA |  |
|  | Probe | FAM-CCGCCGCTCGTCACC-TAMRA |  |
| *Bacteroides* | F | GAGAGGAAGGTCCCCCAC | [6] |
|  | R | CGCTACTTGGCTGGTTCAG |  |
|  | Probe | FAM-CCATTGACCAATATTCCTCACTGCTGCCT-TAMRA |  |
| *Roseburia spp. (SYBR Green)* | F | TACTGCATTGGAAACTGTCG | [7] |
|  | R | CGGCACCGAAGAGCAAT |  |
| *Clostridium perfringens* | F | CGCATAACGTTGAAAGATGG | [8] |
|  | R | CCTTGGTAGGCCGTTACCC |  |
|  | Probe | FAM-TCATCATTCAACCAAAGGAGCAATCC-TAMRA |  |
| *Total bacteria* | F | CGTCAGCTCGTGYCGTGAG | [9] |
|  | R | CGTCRTCCCCRCCTTCC |  |
|  | Probe | TTAAGTCCCRYAACGAGCGCAACCC |  |

Table 3 The detailed information about the enzyme system of the Taqman qPCR

| Reagent | volume | Final concentration |
| --- | --- | --- |
| Sterile water | 6 μL | - |
| KAPA PROBE FAST qPCR Master Mix  10 µM F’ Primer | 10 μL  0.4 μL | 1×  100-400 nM |
| 10 µM R’ Primer | 0.4 μL | 100-400 nM |
| 10 µM Probe | 0.8 μL | 100-500 nM |
| Template DNA | 2 μL | ＜250 ng |
| 50× ROX Low | 0.4 μL | 1× |

Table 4 The sample numbers for each test of faecal metagenomic, serum SCFA analysis and faecal qPCR

|  | Control – Baseline (Day 0) | Control – Endpoint (Day 45) | Oat – Baseline (Day 0) | Oat –Endpoint (Day 45) | Total |
| --- | --- | --- | --- | --- | --- |
| **Metagenomic analysis** | 43 | 43 | 47 | 44 | 177 |
| **Serum SCFA analysis** | 44 | 43 | 47 | 46 | 180 |
| **qPCR** |  |  |  |  |  |
| *Bifidobacterium* | 41 | 39 | 41 | 41 | 162 |
| *Lactobacillus* | 40 | 35 | 38 | 41 | 154 |
| *Akkermancia muciniphila* | 40 | 35 | 40 | 41 | 156 |
| *Roseburia* | 36 | 31 | 36 | 36 | 139 |
| *Bacteroidaceae* | 39 | 34 | 38 | 36 | 147 |
| *Faecalibacterium prausnitzii* | 35 | 29 | 36 | 37 | 137 |
| *Enterobacteriaceae* | 40 | 35 | 39 | 40 | 154 |
| *Clostridium perfringens* | 18 | 14 | 17 | 15 | 64 |

Table 5 : Nutrients intake before and after the interventions in control and oat group

|  | Baseline | | |  | End | | |
| --- | --- | --- | --- | --- | --- | --- | --- |
|  | Control Group | Oats Group | P |  | Control Group | Oats Group | P |
| Energy（kcal） | 1742.45（1615.73，1966.79） | 1723.84（1596.16，1881.04） | 0.333 |  | 1477.02*（1376.53，1604.78） | 1727.52（1556.39，1901.76） | 0.000 |
| Protein（g） | 74.66（66.56，88.41） | 72.56（63.15，85.72） | 0.400 |  | 61.75*（53.33，75.30） | 78.52（60.64，89.14） | 0.000 |
| Fat （g） | 48.05（43.27，54.63） | 49.35（43.83，56.99） | 0.447 |  | 43.45*（39.87，49.06） | 50.61（44.99，57.08） | 0.000 |
| Carbohydrate（g） | 258.98（237.23，289.91） | 251.93（235.58，272.72） | 0.104 |  | 213.13*（192.15，239.08） | 244.39（227.76，274.10） | 0.000 |
| cholesterol（mg） | 249.15（161.57，354.11） | 242.93（154.10，364.65） | 0.674 |  | 233.31（145.31，365.65） | 230.46（140.07，316.87） | 0.612 |
| Fiber（g） | 4.58（2.62，8.54） | 5.31（2.20，8.54） | 0.900 |  | 3.28*（1.57，6.28） | 7.21*（5.47，10.25） | 0.000 |

Supplemental files - Figure 1 The top 15 relative abundance bacterium in each group based on genus

Supplemental files - Figure 2 The top 15 relative abundance bacterium in each group based on phylum

Supplemental files - Figure 3 The α-diversity between groups and days (A: richness; B: Shannon; C: Simpson; D: Peilou)

Supplemental files - Figure 4 The NMDS analysis for β-diversity between groups and days

We performed non-metric multidimensional scaling (NMDS) analysis to explore dissimilarities in the compositional structure of microbiota between the different groups. There was no influence on overall structure of gut microbiota after interventions, and subsequently confirmed by Anosim analysis (P=0.957).

Supplemental files - Figure 5 The NMDS analysis for β-diversity between groups and days

**References**

1 Savino F, Garro M, Montanari P, Galliano I, Bergallo M. Crying Time and RORγ/FOXP3 Expression in Lactobacillus reuteri DSM17938-Treated Infants with Colic: A Randomized Trial. The Journal of pediatrics 2018;192:171-7.e1.

2 Haarman M, Knol J. Quantitative real-time PCR analysis of fecal Lactobacillus species in infants receiving a prebiotic infant formula. Applied and environmental microbiology 2006;72:2359-65.

3 Png CW, Lindén SK, Gilshenan KS, Zoetendal EG, McSweeney CS, Sly LI, et al. Mucolytic bacteria with increased prevalence in IBD mucosa augment in vitro utilization of mucin by other bacteria. The American journal of gastroenterology 2010;105:2420-8.

4 Machiels K, Joossens M, Sabino J, De Preter V, Arijs I, Eeckhaut V, et al. A decrease of the butyrate-producing species &lt;em&gt;Roseburia hominis&lt;/em&gt; and &lt;em&gt;Faecalibacterium prausnitzii&lt;/em&gt; defines dysbiosis in patients with ulcerative colitis. Gut 2014;63:1275.

5 Tuomisto S, Karhunen PJ, Pessi T. Time-dependent post mortem changes in the composition of intestinal bacteria using real-time quantitative PCR. Gut Pathogens 2013;5:35.

6 Zwielehner J, Lassl C, Hippe B, Pointner A, Switzeny OJ, Remely M, et al. Changes in human fecal microbiota due to chemotherapy analyzed by TaqMan-PCR, 454 sequencing and PCR-DGGE fingerprinting. PloS one 2011;6:e28654.

7 Bergström A, Licht TR, Wilcks A, Andersen JB, Schmidt LR, Grønlund HA, et al. Introducing GUt Low-Density Array (GULDA) – a validated approach for qPCR-based intestinal microbial community analysis. FEMS Microbiology Letters 2012;337:38-47.

8 Just NA, Létourneau V, Kirychuk SP, Singh B, Duchaine C. Potentially Pathogenic Bacteria and Antimicrobial Resistance in Bioaerosols from Cage-Housed and Floor-Housed Poultry Operations. The Annals of Occupational Hygiene 2012;56:440-9.

9 Wang Y, Qian P. Conservative Fragments in Bacterial 16S rRNA Genes and Primer Design for 16S Ribosomal DNA Amplicons in Metagenomic Studies. PloS one 2009;4:e7401.
